# Advanced Membrane Technologies for Wastewater Treatment and Recycling

**DOI:** 10.3390/membranes13060558

**Published:** 2023-05-29

**Authors:** Hongjun Lin, Meijia Zhang

**Affiliations:** College of Geography and Environmental Sciences, Zhejiang Normal University, Jinhua 321004, China

In the face of the ever-growing severe problem of water scarcity, wastewater reuse, recycling and resource recovery are increasingly recognized as crucial part of the solution. Compared to other wastewater treatment processes, membrane technology stands out with its distinctive advantages, including simple operation, easy scalability, and chemical-free operations, and has, therefore, been extensively employed in wastewater treatment and recycling. Despite the significant progress in implementing membrane technologies for wastewater treatment and recycling, the application of membranes in wastewater treatment still confronts various unresolved challenges, such as membrane fouling.

This current Special Issue on *Membranes* aims to comprehensively cover state-of-the-art advancements and future developments in the field of membrane technologies applied to wastewater treatment and recycling. Following the proposal for this Special Issue, seventeen research articles and one communication were published, all of which contributed significantly to the scientific understanding of this field. This article presents a concise summary of the research articles included in this Special Issue.

Half of the published papers in this Special Issue focused on membrane fabrication and modification, with new strategies developed to improve membrane separation [1,2,3,4], permeability [5,6,7], and anti-fouling properties [8,9] by fabricating the membrane structure or surface properties. For example, Fang et al. [1] fabricated a loose nanofiltration membrane by integrating blending and interfacial coordination strategies; this resulted in a membrane with good pure water flux, dye rejection, and salt penetration. Liu et al. [5] synthesized a covalent–organic framework (COF) composite membrane by assembling COF layers and the imidazole-quartet water channel, which exhibited excellent performance above 271.7 L m^−2^ h^−1^ bar^−1^ water permeance and above a 99.5% congo red rejection rate. Wang et al. [9] improved the antifouling performance of this membrane by modifying the membrane surface hydrophilicity and porosity by mixing polycationic liquid into the polyvinylidene fluoride (PVDF) membrane.

Of the remaining eight articles, half were related to the application of membrane bioreactors in wastewater treatment. Dong et al. [10] optimized the membrane biofilm reactor and hydrogen-based membrane biofilm reactor (MBR-MBfR) to treat low C/N wastewater and found that proper system functioning was achieved by coupling the partial nitrification-denitrification (PN-D) process in an MBR with further treatment in an MBfR. Aguilar-Moreno et al. [11] demonstrated the economic feasibility of ammonia recovery from anaerobic digestion concentrate using a combination of C/F, aeration, and membrane contactor. Zou et al. [12] reported that the MPBR system could not maintain long-term operations under high SRT for municipal wastewater treatment. Such operating conditions may lead to the decay and deterioration of MPBR’s biological performance while improving the antifouling performance of microalgae flocs. Alharthi et al. [13] successfully integrated the moving bed biofilm reactor (MBBR), membrane bioreactor (MBR), and direct contact membrane distillation (DCMD) treatment steps for industrial wastewater treatment, and the results showed that high-quality effluents were obtained by the three-step process.

Cunha et al. [14] characterized water dynamics in cellulose acetate-silica asymmetric membranes by combining multiple methods, including ^1^H NMR spectroscopy, diffusometry and relaxometry. In the research conducted by Lu, Bai, and Liao [15], mathematical modeling was utilized to examine the impact of temperature (mesophilic versus thermophilic) and oxygen partial pressure on the performance of the membrane-aerated biofilm reactor (MABR), and the results indicated that ThMABR had significant advantages over conventional mesophilic MABR. Zou et al. [16] investigated the role of Ca^2+^ addition in humic acid (HA) fouling and the potential of adding Ca^2+^ for fouling mitigation in the coagulation-ultrafiltration process. The results demonstrated the feasibility of fouling mitigation by adding Ca^2+^ into the ultrafiltration process to treat HA pollutants. Li et al. [17] investigated the water purification effect and membrane fouling mechanism of two types of powdered activated carbon (PAC) that enhanced PVDF ultrafiltration membranes for surface water treatment. The results showed that PAC could effectively enhance membrane filtration performance.

Pereira et al. [18] reported on a study that evaluated the applicability of a previously developed mathematical model to predict the fractionation of aromas from different chemical families in real effluents (sardine cooking wastewaters) and remove off-flavors. Their findings demonstrated that the model simulations were not substantially impacted by the food matrix, which served to validate and expand the applicability of the model.

The field of advanced membrane technologies for wastewater treatment and recycling encompassed a broad range of research topics. This Special Issue presents significant contributions to membrane research, covering membrane fabrication and modification, membrane bioreactor applications in wastewater treatment, and membrane fouling control and mechanisms. In conclusion, the editors appreciate the authors’ and reviewers’ valuable contributions to this Special Issue. We are also grateful to the editorial staff of “Advanced Membrane Technologies for Wastewater Treatment and Recycling” for their invaluable support.

## Data Availability

Not applicable.
